# The use of cyanoacrylate in the treatment of angiodysplasias: A safe and cheap alternative to coils

**DOI:** 10.1259/bjrcr.20210130

**Published:** 2022-09-12

**Authors:** Pietro Pitrone, Alberto Stagno, Antonino Cattafi, Simona Caloggero, Salvatore Silipigni, Velio Ascenti, Francesca Catanzariti, Antonella Cinquegrani, Antonio Bottari

**Affiliations:** 1 Department of Biomedical and Dental Sciences and of Morphological and Functional Images, Section of Radiological Sciences, University of Messina, Messina, Italy; 2 Department of Imaging, Interventional Radiology Unit, University Hospital “G. Martino”, Messina, Italy; 3 Postgraduate School of Diagnostic and Interventional Radiology, University of Milan, Milan, Italy

## Abstract

Gastrointestinal angiodysplasia (GIAD) represents one of the most frequent causes of recurrent lower gastrointestinal bleeding in the elder population. Clinical manifestations are highly variable, diagnosis is done with colonoscopy or CT and management consists of either endoscopic or, more conservatively, endovascular approach. Trans-arterial embolization (TAE) reduces blood flow into the lesion and may complicate with perforation, dissection, vasospasm and bowel ischaemia. To date, coils and Gelfoam represent the most employed embolizing agents, followed by PVA and onyx. We report the successful embolization of GIADs in four patients with n-butyl 2-cyanoacrylate (NBCA) and Lipiodol Ultra-Fluid (LUF): despite the reported higher risk of bowel infarction when compared with the other agents, no major complication or short-term recurrence occurred in our series.

## Summary

Gastrointestinal angiodysplasia represents a common vascular GI lesion^1^ and a frequent cause of recurrent and obscure bleeding.^2^ Elder population is mostly involved, with no sex prevalence,^3^ and cecum and ascending colon are usually affected.^1^ Intermittent obstruction of submucosal veins during muscular contraction and caecum distension with secondary ischaemia and neoangiogenesis are advocated.^2^ Clinical manifestations include maroon-colored stool, melena, hematochezia, iron deficiency anaemia or/and occult blood within faeces.^1,2,4^ Diagnosis is done by endoscopy (mainly upper and colonic lesions), allowing interventions but needing bowel preparation and CT-angiography, detecting active or recent extravasations throughout the whole GI tract^5^; although active extravasation is rarely visible, DSA-angiography shows well the anatomy of the vascular malformation.^6,7^ Whenever conservative treatment^8^ fails, endoscopic or endovascular treatment is performed, the latter consistent in TAE, aiming to reduce blood flow towards the lesion: coils and gelfoam are the most employed agents, followed by PVA particles, onyx and, lastly, N-butyl 2-cyanoacrylate.^1,6^ Despite the higher risk of reflux and bowel ischaemia when compared to coils, we hereby describe a four cases-experience of TAE with Glubran and suggest a larger use of the latter, due to the low cost and high efficacy whenever appropriate management of the agent/catheter is taken.

## Clinical presentation

**Figure 1.**
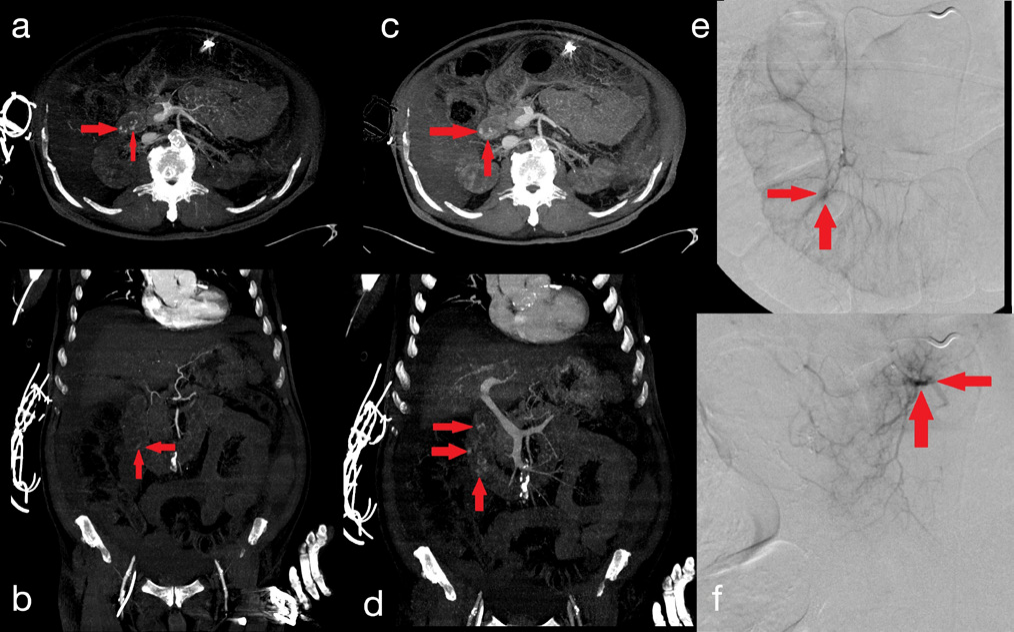
a 79 years old male presenting with recurrent melena, abdominal pain and anaemia. Contrast-enhanced CT scans of the abdomen show an active extravasation of contrast medium within duodenal lumen, incrementing from the arterial (**A-B**) to the venous phase of the study (C-D; red circles). DSA is thus performed: the presence of an active bleeding from a branch of the gastro-duodenal artery is confirmed (E; red circle) and successful TAE with 2 ml Glubran 60% is performed (**F**).

**Figure 2.**
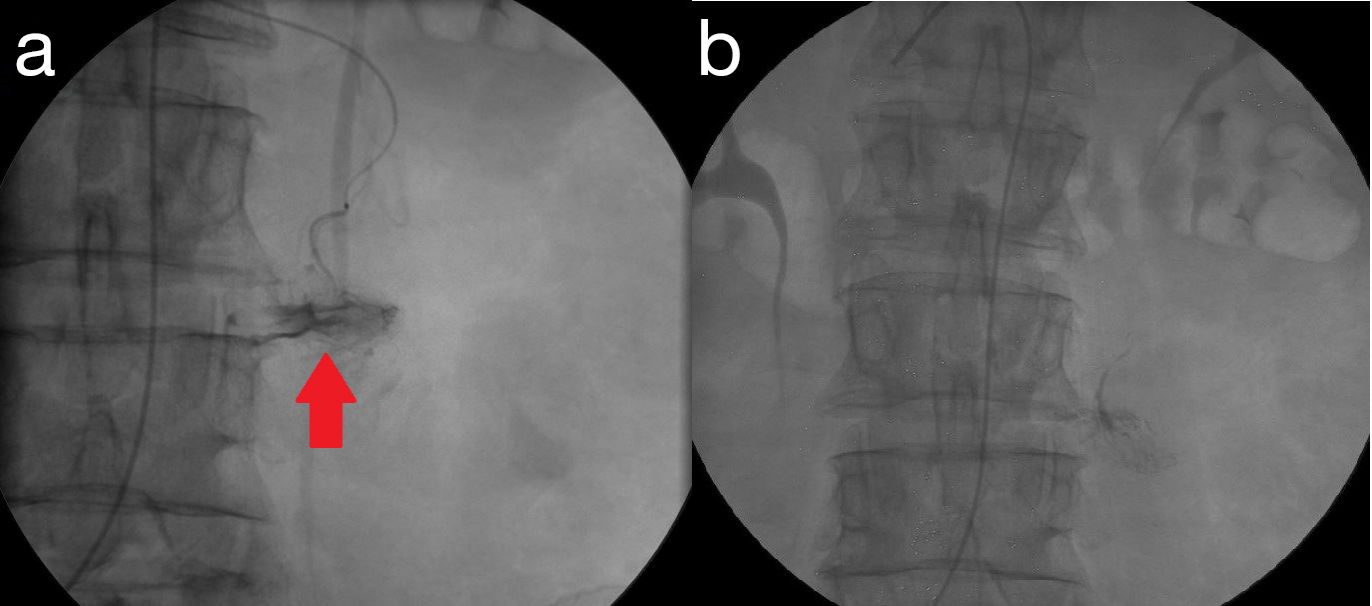
a 73 years old female presenting with melena and anaemia. CT-angiography of the abdomen does not show any sign of active or recent bleeding. DSA demonstrates the presence of an active bleeding from jejunal branches of the superior mesenteric artery (A; red arrow) and successful TAE with 2 ml Glubran 40% is performed (B; the radiopacity within the feeding artery represents Glubran-Lipiodol mixture [the second being iodine-rich], which has stopped blood flow into the “vascular tufts” [no longer opacified]).

**Figure 3.**
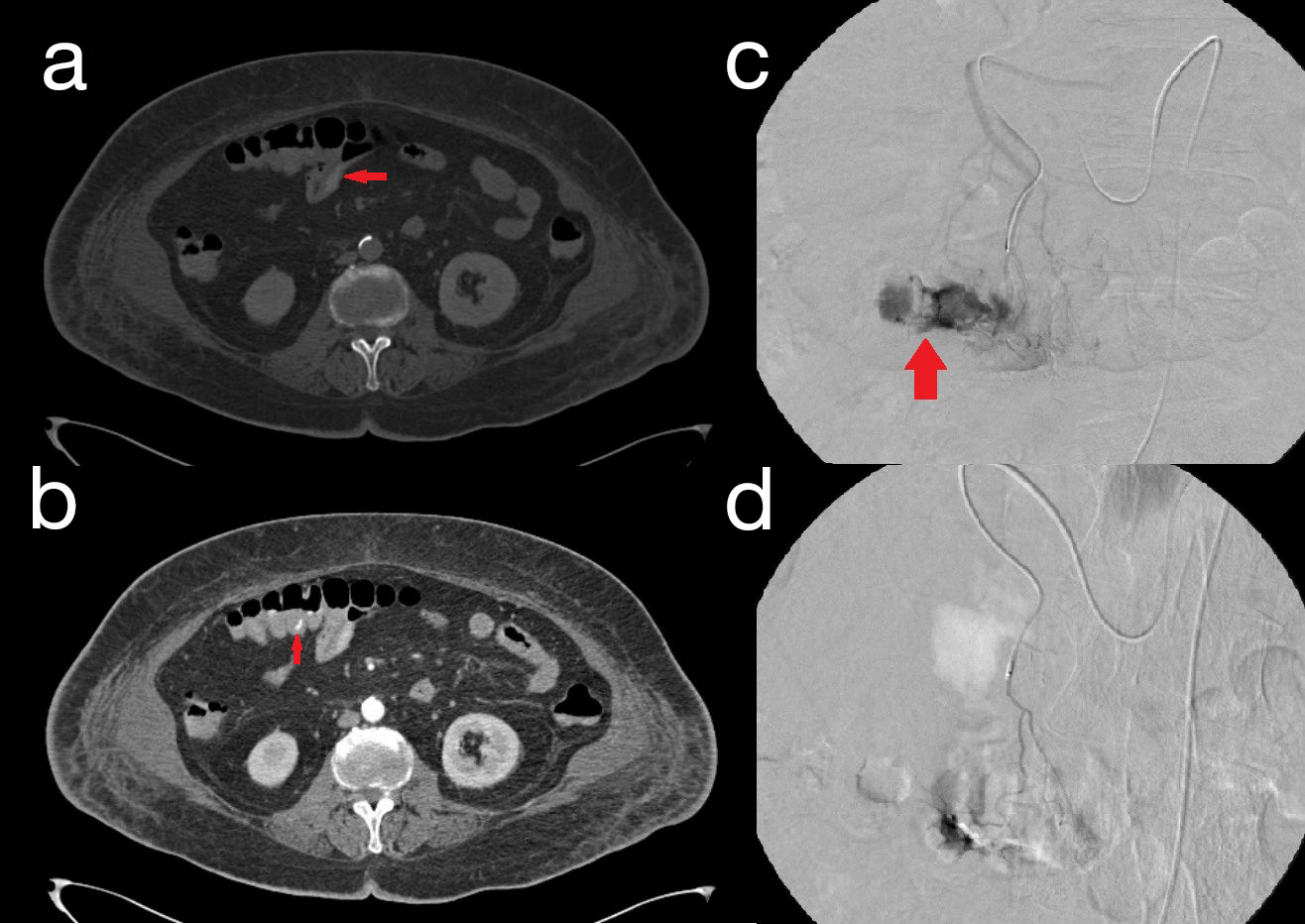
a 72 years old female presenting with rettorrhagy and anaemia. Pre (**A**) and post-contrast CT scans of the abdomen (**B**) show clots and active extravasation of contrast medium within transverse colic lumen, respectively (red arrows). DSA is thus performed: the presence of an active bleeding from a branch of the middle colic artery is confirmed (C; red arrow) and successful TAE with 2 ml Glubran 30% is performed (**D**).

Four patients (three female: one male; mean age: 79.25 years; range: 72–93 years) with recurrent melena/rettorrhagy and anaemia were admitted to Our Centre, nearly all presenting several risk factors like cardiopathy, hypertension, diabetes and liver/renal failure. One patient (79, M: Figure 1) already showed endoscopically diagnosed duodenal angiodysplasia in anamnesis; one (93, F) had suspected colonic angiodysplasia due to recurrent episodes of hematochezia over time (negative colonoscopy); one (73, F: Figure 2) had suspected duodenal/jejunal angiodysplasia due to persistent anaemia (negative gastro-duodenal endoscopy); the last one (72, F: Figure 3) had suspected colonic angiodysplasia due to massive hematochezia which represented a contraindication for washing and, thus, endoscopy (Table 1).

**Table 1.** details from the patients included in our study

Patient	Clinical presentation	CT findings	DSA findings	Embolizing agents
79 years old male (Figure 1)	Recurrent melena with anaemia, diffuse abdominal pain, decompensated cirrhosis, cardiopathy, hypertension, diabetes, renal failure. Known duodenal angiodysplasia in anamnesis (previous endoscopies)	Duodenal *blush* of contrast medium on enhanced scans	gastro-duodenal artery active bleeding (duodenal branch)	Glubran:LUF 60–40% (2 ml)
93 years old female	Rettorrhagy with anaemia, cardiopathy, hypertension. Suspected colic angiodysplasias due to recurrent episodes of hematochezia over time (negative colonoscopy)	Transverse colic *blush* of contrast medium on enhanced scans	Middle colic artery active bleeding (ascending branch)	Glubran:LUF 50–50% (1 ml) and coils (2 × 25 mm)
73 years old female (Figure 2)	Melena with anaemia. Suspected jejunal/ileal angiodysplasias due to persistent anaemia (negative duodenal endoscopies)Duodenal *blush* of contrast medium on enhanced scans	No significant *blush* (probably under CT threshold)	Superior mesenteric artery active bleeding (jejunal branches)	Glubran:LUF 40–60% (2 ml)
72 years old female (Figure 3)	Rettorrhagy with anaemia, cardiopathy, hypertension, diabetes, renal failure, chronic cerebral vasculopathy. Suspected colic angiodysplasias (massive hematochezia)	Transverse colic blood *clots* on unenhanced scans	Middle colic artery active bleeding	Glubran:LUF 30–70% (2 ml)

## Investigations/Imaging findings

Patients were transferred to Our Department of Radiology and CT-angiography was performed in order to detect signs of active or recent GI bleeding responsible for anaemia in subjects with already diagnosed or clinically suspected angiodysplasia or to seek for any other potential GI pathological condition. Hyper-dense “*blushes*”/*clots* in duodenal or colic lumen on enhanced/unenhanced scans respectively were detected in all but one patient; despite the negativity of CT the Operator, given the clinical suspicion and in agreement with the colleagues from the Emergency Department and the patient, performed DSA in order to detect potential sources of low-rate bleeding. Via a right common femoral artery access with a 5 Fr sheath (Cordis [Santa Clara, Calif] or Terumo [Tokyo, Japan]), a 0.035 inch. hydrophilic guide-wire (Terumo) and a 4–5 Fr hydrophilic catheter (Cobra C2 or Sim 1, Cordis), selective angiography of gastro-duodenal/superior mesenteric artery was obtained (15 ml at 4 mL/sec and 50–60 ml at 6–8 mL/sec, respectively),followed by super-selective angiography of branching vessels (3–4 ml s^−1^) through a co-axial 2.5–2.8 Fr micro catheter (Maestro [Merit Medical, South Jordan, UT], Asahi [Asahi Intecc, Nagoya, Japan] or Cantata [Cook Medical, Bloomington, IN]) with straight design, advanced on micro-guidewires with modifiable tips but “memory properties”. Active extravasation of contrast medium from arteriovenous malformation with vascular tufts within gastro-duodenal, middle colic and jejunal arteries/branches was found; thus, angiographic diagnosis of angiodysplasia was confirmed in one patient and made in 3 patients with a high confidence level.

## Treatment, outcome, follow-up

Once target vessels were identified on DSA and the micro catheter was placed with the tip close enough to the source of bleeding (in order to grant collateral re-vascularization and avoid ischaemia of the bowel tract), TAE with Glubran was performed. To the knowledge, Glubran is a non-absorbable and non-radiopaque synthetic surgical glue with haemostatic, adhesive, sealer, and bacteriostatic properties; when in contact with anionic substances (that is, in moist environments [plasma, blood cells, endothelium, saline]), it quickly polymerizes into a thin elastic film which has high tensile strength and firmly adheres to the anatomy of the tissue on which it is applied. In particular, a variable dilution with LUF (from 30 to 60%, slowly turning syringe upside down) was employed in order to delay polimerization: the closer the source of bleeding, the higher the concentration of cyanoacrylate, also according to the Operator’s experience. Injections of 0.2–0.3 micro boluses, followed by flushes of cold 5% dextrose solution in order to wash micro catheter’s dead space and allow periodic post-contrastographic evaluations, are performed until clear stop of blood flow into the lesions, which occurred after administration of 1–2 ml of solution. Whenever enough cyanoacrylate is injected and few boluses still fill the micro catheter’s dead space, the Operator may prevent non-target embolization by safely removing the catheter while aspirating. Technical success was 100%, with only one patient requiring two additional coils; no major complication such as bowel ischaemia was reported and neither clinical/laboratory recurrence nor endoscopic sign of the malformation was observed within the first two years post-operatively.

## Discussion

Gastrointestinal angiodysplasias, also known as angioectasias, arteriovenous malformations or vascular ectasias, were first described in literature in 1839 and consist of enlarged arteries, veins and “tufts” within bowel wall.^1^ They represent the most common vascular lesion of the GI tract, the second most frequent cause of lower GI bleeding (after diverticula), the first in mid small-bowel tract and a common cause of recurrent, obscure bleeding and anaemia. They usually involve the elder population (accounting for 40% of cases among patients older than 60 years).^1,2^ Both sexes are equally affected.^3^ GIADs are usually multi focal and mostly involve caecum and ascending colon (75%), followed by jejunum and ileum (15%).^1^ According to Boley and Brandt, intermittent obstruction of sub mucosal veins during contraction of muscularis propria and caecum distension leads to congestion of the capillaries and failure of pre-capillary sphincters; the subsequent hypoxaemia leads to the development of small arteriovenous collaterals by the means of increased vascular endothelial growth factor. Some suggested that underlying cardiac, vascular or pulmonary diseases might contribute to mucosal ischaemia from chronic hypoxia or hypo-perfusion and thus AD. No familial, cutaneous or systemic disorder has ever been associated. The result is that of multiple, focal, tortuous, thin-walled vascular dilations within the bowel wall, early draining veins or supplying enlarged artery and vascular tufts.^2^ Clinical presentation varies from absence of symptoms (0.83% of subjects with colonic GIADs), maroon-colored stool, melena (black tarry stools, usually from small bowel or proximal colon), hematochezia (red blood per rectum, from large bowels or acute and massive blood loss) or iron deficiency anaemia and stool intermittently positive for occult blood (being alone in 15% of cases) to massive blood loss (15%).^1,2,4^ Thus, a differential diagnosis with other causes of lower gastrointestinal tract bleedings (that is, below the ligament of Treitz) involving both the large (90%) and the small bowel (10%) is compulsory. Such conditions include, with decreasing frequency, colonic diverticula, vascular (haemangiomas, angiosarcomas) and non-vascular tumours and inflammatory lesions, post-operative bleedings, benign anorectal lesions (such as haemorrhoids), hereditary haemorrhagic telangiectasia, post-radiation vascular ectasias and Dieulafoy’s lesions.^1^ Upper and colonic AD are usually diagnosed by standard endoscopy and colonoscopy, the latter (sensibility 90%, positive predictive value 87%) allowing local interventions but needing bowel preparation and not feasible with massive blood loss^5^; enteroscopy is advocated in the small bowel.^3^ CT angiography (accuracy 80%, sensibility 50–86%, specificity 92–95%, PPV 100%) detects intra luminal blushes of contrast/clots in enhanced/unenhanced scans, indicating active extravasation (with a minimum flow rate of 0.3–0.5 mL/min) and recent bleeding, respectively.^5^ Indirect signs of the presence of a lesion causing haemorrhage are hyper attenuating peri enteric fat, bowel wall thickening and/or enhancement, polypoid lesions and tumour masses.^1^ Angiography of the superior and mesenteric arteries (sensibility 58–86%, specificity 100%, PPV 100%, NPV 24%) needs no bowel preparation. Active extravasation (with a minimum flow rate of 0.5–1 mL/min) is found in 6–20% of cases only^6^; densely opacified, dilated, tortuous and slowly emptying intramural veins, vascular tufts (that is, dilated mucosal venules and capillaries) in the arterial phase and, at the latest stage, arteriovenous communication are specific of AD.^7^ GIADs’ bleeding stops spontaneously in over 90% of cases but is often recurrent.^4^ Conservative treatment includes iron supplements, octreotide and thalidomide^8^; if symptoms of blood loss are present, endoscopic or endovascular treatment is performed.^1,7^ Trans catheter arterial embolization (TAE), first described by Bookstein et al. in 1974, favours clot formation while maintaining collateral flow to avoid bowel infarction. It appears safer than surgical intervention in high-risk patients (mortality 9–47%) and does not preclude the latter. Target vessels are “vasa recta” and marginal or terminal artery when treating the SMA and the IMA, respectively. Coils are permanent agents, range from sub millimetre to centimetres and are made of a metal component which causes physical occlusion and a fibre one stimulating thrombosis, are visible with fluoroscopy (thus being the easiest to deploy and best visible at surgery) and, especially with the newer, can be removed if necessary. The risk of reflux of particles, as well as the risk of bowel ischaemia (since they best allow collateral flow in), are the least, which explains why they are the most used. Control release coils further grant precise deployment. Gelfoam (absorbable compressed sponge) is a temporary (weeks to months) agent made of subcutaneous porcine adipose tissue, often mixed with saline to form a slurry (helping its delivery). It’s cheap, widely available and allows future access to the vessel, but it needs proper preparation, re-canalization is unpredictable and, due to the small size of the particles (2–3 mm are advised), embolization distally, in the nearby collaterals or even reflux in non-target arteries may occur PVA particles (150–250 to 500 micron) show good results but are radio transparent, can embolize non-target vessels or block small microtubules. Onyx is another liquid embolic agent made of ethylene- vinyl alcohol copolymer dissolved in dimethyl sulphoxide (DMSO) well described in 2010 by Lenhart and coll in the treatment of upper GIB, with a success rate of 81% and minimal complications. It is non-adhesive, radiopaque and solidificates in a longer time, which makes the occlusion more predictable, but it often causes vasospasm, is excreted via respiration/perspiration for days, it’s expensive and requires DMSO compatible catheters. N-butyl 2-cyanoacrylate (NBCA) glue is a permanent agent, polymerizing when exposed to blood, normal saline or any other ionic solution; nevertheless it can embolize, since it’s mixed with Lipiodol (ratio usually 1:2-1:3, depending on the distance from the source of bleeding), beyond the tip of the micro-catheter (0.01 inches), especially in very small calibre vessels, thus achieving distal occlusion, which reduces the chance of bleeding from collateral circulation. It grants fast and permanent closure (independent of the thrombose ability of the patient and with rebleeding percentage of 4–15% only), can manage complex pseudo aneurysms and can be delivered through micro catheters non-suitable for coils. Its super selectivity allows the Operator to embolize arteriovenous fistula, arterial vessel and drainage vein at the same time.^6^ Thanks to these features, NCBA has been previously and successfully employed with symptomatic renal angiomyolipoma^9^; there is also witness of extravascular applications, as in the case of bilio-cutaneous fistulas.^10^ However, it requires a high operator experience, shows a higher risk of agent reflux, bowel infarction and future stenosis and, due to the polymerization within the catheter, can make it become adherent to the artery. To avoid that, a non-ionic agent like dextrose solution (usually 5%) must be employed to initially wash the micro catheter and then pull all injected NCBA out of the former and prompt removal of the micro catheter is advised^6^ ; also, syringes previously containing glue should be dropped after embolization. Zhao (2016) successfully performed TAE with cyanoacrylate in seven patients with chronic repeated GI haemorrhage failing conservative treatment; only one (14%) experienced rebleeding (due to the presence of a second straight artery supplying the lesion) and no one suffered from bowel ischaemia or necrosis.^8^ To date, micro coils and sponge represent the most used agents.^1^ Success rates reach 80–100%; failure (20%) can be secondary to vessel perforation, dissection, spasm, cessation of bleeding or vessel tortuosity. Early (<30 days: 10–30%) o delayed (>30 days: 7.69%) rebleeding may occur due to re-canalization of previously embolized vessels or new sources of bleeding.^8^ Common complications (5–9%) include haematomas, thrombosis, pseudo aneurysm formation and bowel infarction^3^; lower GIADs present a higher risk due to the less developed artery anastomosis network, especially with history of GI surgery/radiotherapy.^8^ In conclusion, TAE represents the gold standard in the treatment of GIADs, especially within the small bowel or in case of heavily bleeding colic lesions. Despite the lowest risk of recurrence, non-target embolization or bowel necrosis, coils are expensive and require compatible catheters. Few experiences in literature are reported about the use of NBCA due to the higher risk of reflux and bowel ischaemia. However, it is cheap, does not require specific micro catheters, shows strong, permanent and patient-independent efficacy. Of course, as in any other delicate endovascular procedure involving gastrointestinal tract, the Operator experience (especially when diluting glue and flushing catheters), plays an important role. Thus, patients should be managed within an Interventional Radiology Unit and confrontation with surgeons prior to DSA and after the last diagnostic acquisition before TAE is advised in order to evaluate potential clinical implications and risks (*e.g.,* bowel ischaemia/necrosis). Our series included a limited number of patients diagnosed with GIADs due to the evanescent nature of these bleedings. Although none of our patients experienced bowel necrosis or rebleeding, further and large-scale studies are necessary to confirm the feasibility of NCBA with GIADs.

## Informed consent statement

Written informed consent for the case to be published (incl. images, case history and data) was obtained from the patient for publication of this case report, including accompanying images.

## Learning points

- Gastrointestinal angiodysplasia (GIAD) is the second most frequent cause of lower GI bleeding in the elder population and is usually recurrent and clinically unnoticed.
- Diagnosis is made by the means of colonoscopy, allowing local interventions but needing preparation and unadvisable in case of massive blood leakage, and CT-angiography, offering a panoramic view of the GI tract and the possible cause of bleeding.
- Endoscopic or endovascular management are advised only in case of symptoms of blood loss.
- Trans-arterial embolization (TAE) reduces blood flow into the lesion thus favouring clot formation, while adequate collateral flow is granted distally through alternative vessels in order to avoid bowel infarction; despite the risks (haematomas, thrombosis, pseudo aneurysm formation, perforation, dissection, vasospasm and bowel infarction), TAE is safer than surgery.
- To date, the most used agents remains coils, for the minimal risk of reflux and bowel necrosis, and sponge, for the low cost and large availability; few experience is documented about the use of PVA particles and onyx.
- N-butyl 2-cyanoacrylate (NBCA) with Lipiodol is cheap, feasible with any catheter, fast-acting, long-lasting, independent of the thrombose ability of the patient and reduces the risk of collateral bleeding. The main issues remain the need for a high operator experience, due to the risk of polymerization within the catheter or non-target embolization and, most importantly, bowel infarction. However, a careful dilution with Lipiodol for a precise administration, quick washing with non-ionic solutions (*e.g.,* 5% dextrose) and catheter removal manoeuvrers can lower such risk and elevate NBCA as one of the agents of choice in the TAE of GIADs, as witnessed by Our experience. In this setting, a cooperation between an Interventional Radiology and a Surgery Unit might assist in a risk-benefit analysis prior to embolization.
